# Building the Electromagnetic Situation Awareness in MANET Cognitive Radio Networks for Urban Areas †

**DOI:** 10.3390/s22030716

**Published:** 2022-01-18

**Authors:** Paweł Skokowski, Krzysztof Malon, Jerzy Łopatka

**Affiliations:** Institute of Communications Systems, Faculty of Electronics, Military University of Technology, Gen. Sylwester Kaliski Str. No. 2, 00-908 Warsaw, Poland; krzysztof.malon@wat.edu.pl (K.M.); jerzy.lopatka@wat.edu.pl (J.Ł.)

**Keywords:** spectrum monitoring, data fusion, the decision-making process, cognitive radio network, MANET, dynamic spectrum management, ad hoc networks, machine learning

## Abstract

This paper presents a solution for building awareness of the electromagnetic situation in cognitive mobile ad hoc networks (MANET) using the cooperative spectrum sensing method. Signal detection is performed using energy detectors with noise level estimation. Based on the evidence theory, the fusion center decides on the particular channel occupancy, which can process incomplete and unambiguous input data. Next, a reinforced machine learning algorithm estimates the usefulness of particular channels for the MANET transmission and creates backup channels list that could be used in case of interferences. Initial simulations were performed using the MATLAB environment, and next an OMNET-based MAENA high fidelity simulator was used. Performed simulations showed a significant increase in sensing efficiency compared to sensing performed using simple data fusion rules.

## 1. Introduction

Self-organizing mobile ad hoc networks (MANETs) are modern and flexible cognitive solutions working without controlling infrastructure. Usually, they do not use the Primary User and Secondary User paradigm, but all radio nodes have the same rights, and all nodes compete for the spectral resource. That is why spectrum monitoring is an essential element of radio’s cognitive cycle used to assess the current spectral situation [1]. Furthermore, the quality and accuracy of the achieved data are crucial for the radio network operation parameters. This work aims to propose a solution devoted to MANETs working in the presence of interference and intentional jamming. Such an assumption eliminates the possibility of wideband monitoring because high-level interferences may block the wideband receiver. Furthermore, full-duplex sensing is also inefficient because fast-changing conditions of signals reception disable efficient iterative adaptation of self-interference cancelation algorithm [2,3,4,5].

On the other side, the spectrum-monitoring results’ reliability depends on the signal detector type, detection time, assumed detection threshold, and detection periodicity [6]. Frequencies may be controlled periodically, randomly, or according to predefined priorities to optimize the last parameter. Both spectrum detectors, detection strategy, and data fusion methods should be appropriately selected to achieve the optimal results according to the predefined goals and policy. The sensing strategy dilemma is related to the optimal amount of time devoted to spectrum monitoring because, during time slots dedicated to spectrum monitoring, data transmission is usually suspended to avoid interferences from own transmitters, so longer and more frequent slots assigned to the spectrum monitoring, lead to limitations of the throughput available to the user.

The sensing results are used in MANET for agile spectrum utilization, and these methods may be divided into two major groups: reactive and proactive. Reactive strategies enable the adaptation of used spectral resources based on searching the newly available resource after the used resource is jammed or interfered with. In contrast, the proactive approach is based on the sequential wideband search of idle resources that can be used as a backup in the case of interference. Such monitoring is performed periodically during the network operation and enables faster network reconfiguration in case of interference interrupting the communication. Mainly, if possible, backup channels are distributed within the network in advance [7]. However, the major disadvantage of such an approach is the need to introduce silence periods in transmission for the detection process. Furthermore, additional time for sensing control and distribution is required.

In MANET, spectrum sensing may be performed in a distributed or cooperative manner. In distributed methods, each node assesses the spectrum only from its point of view, whereas in a corporative way, nodes exchange information about spectrum occupancy between them. Because radio spectrum is a multi-dimensional space described by geographical coordinates, frequency, and time, its properties depend on the observation point and detection method. Apart from this, propagation conditions play a crucial role in spectrum assessment by specific nodes because terrain shape and obstacles may suppress radio signals at specific locations and disable their detection, and this is the main reason why cooperative spectrum sensing is widely used [8,9,10,11].

The simplest and the most popular spectrum detector is the energy detector. However, spectrum analysis can work as a single channel detector or multi-channel one, and it doesn’t need to know the detected signal structure [12]. According to [9,10,12,13,14,15], apart from classical energy detector, cumulative power spectral density detector, cyclo-energy detector, and generalized energy detector may be used. Moreover, known user’s signal pattern may be detected by cyclo-stationary, auto-correlation, and eigenvalue-based detectors. Another approach is pilot-based user’s signal waveform detection by the matched filter, knowing the detected signal structure [10].

The sensing strategy dilemma is related to the optimal amount of time devoted to spectrum monitoring because, during time slots dedicated to spectrum monitoring, data transmission is suspended to avoid interferences from own transmitters, so longer and more frequent slots assigned to the spectrum monitoring lead to limitation of the throughput available to the user.

A cooperative spectrum monitoring may be used, where several radios perform spectrum monitoring. According to predefined fusion rules, radio channel occupancy’s decision is taken in the Fusion Center (FC). Data fusion may be performed using soft combining, quantized, or hard combining [16]. The first approach assumes that all results from the local sensing are available at the FC. Usually, equal gain combining (EGC), maximal ratio combining (MRC), LRT based or modified deflection coefficient (MDC) methods are used. Such an approach enables optimal conditions for decision making in FC. Still, the transmission of many sensing-related data must be transmitted, so this solution may be used mainly in wideband systems for slowly changing spectrum conditions. The Quantized combining limits the transmission needs by locally performed strong data selection and compression to several bits, leading to sub-optimal FC decisions.

To improve the fusion efficiency, in [8], authors proposed to use Markov Random Fields that approximate users’ spectrum statuses before combining the particular results. Additionally, neighbor nodes likely have the same spectrum status, enabling detection reliability or minimizing control traffic by eliminating neighbor nodes from the sensing procedure. Another approach is presented in [11], where the residual neural network is combined with feature extractor and random forest classifier. The feature extractor reduces the signals’ complexity and speeds up the response time. Authors claim that the achieved results are promising, but the number of extracted features must be reduced for resource optimization. Moreover, the proposed approach assumes the existence of primary and secondary users that is not the case for military MANET.

To futher minimize the control traffic, compressed sensing with the hard decision is proposed in [17]. Two decision thresholds are defined to enable three possible choices: signal absent, signal present, and uncertain state. When the signal is absent, no sensing results are transmitted. It limits the number and size of control messages but leads to ambiguous situations where the FC doesn’t know if the results are unavailable or signals are absent. Therefore, it may lead to wrong decisions about spectrum occupancy. Moreover, proposed ‘DOR’ fusion is a modification of the well-known ‘OR’ rule and generates a relatively high level of false alarms [9].

Spectrum occupancy of different communication systems is closely related to their structure, used modulation and coding scheme, medium access control, link layer, transport layer, and used applications. It means that in some cases (e.g., for broadcasting services), frequencies are occupied or idle for relatively long periods, but in most cases, their status is frequently changing, e.g., from message to message. In addition, because of node mobility and multipath propagation, signal detection may be impossible from time to time. Therefore, it means that periodically detected spectrum occupancy may change very often, and the assessment of the usefulness of specific frequencies for radio transmission should be additionally performed.

In this paper, the authors propose an integrated solution for spectrum awareness containing both spectrum monitoring and radio channel utility evaluation (RCUE). The spectrum monitoring uses energy detection with noise level estimation and FC rules based on the Dempster–Schaffer evidence theory, designed for reasoning based on incomplete, imprecise, and uncertain information. The authors presented its details in [18] and achieved results showing that it provides a high probability of signal detection, maintaining a low level of false alarms. Next, the authors propose an additional processing step—RCUE. The solution is based on artificial intelligence, using reinforcement learning to calculate the utility of particular radio channels and enables reliable estimation of the usefulness of specific frequency channels for MANET use. Authors describe its details in [19].

The structure of this paper is as follows: Section 2 is devoted to the general description of the proposed spectrum monitoring solution, Section 3 presents solution evaluation using both MATLAB and the MAENA Simulator, whereas Section 4 contains conclusions.

## 2. Cooperative Spectrum Monitoring Using Data Fusion and Machine Learning

### 2.1. Cooperative Spectrum Monitoring

Detection of radio spectrum must cope with many drawbacks occurring in the radio environment, such as shadowing or the hidden node problem [1,9,20], which for the Cognitive Radio Network (CRN) user translates to limited ability to sense signal effectively. Cooperation between Cognitive Radio (CR) nodes is one of the most effective methods to deal with those problems. The solution is to make decisions based on reports received from several CR nodes. Based on the information received from many radio nodes, a decision whether a signal is present or not may be taken, even if some of the CRN nodes could not correctly detect occurring transmission (e.g., due to the large distance from the signal source or shadowing effect).

Considering the previous works [21,22] and the solution from [23], a cluster-based MANET architecture is proposed. MANET is divided into groups of nearly located nodes, called clusters. Each cluster is managed by an elected Cluster Head (CH). All CR nodes perform their local detections during spectrum monitoring using an energy detector with estimated noise power (ED-ENP) [14,15]. The sensing performance is strictly dependent on the used threshold in terms of the probability of detection ( $P_{d}$) and false alarm probability ( $P_{fa}$). The threshold has to be set according to the channel condition and the noise power at the receiver. Its estimation is critical in spectrum sensing [14,24], particularly energy detection methods. If the noise power is known and the number of samples is not fixed, it is theoretically possible to choose a threshold that can simultaneously meet any target $P_{d}$ and $P_{fa}$ [14,25].

The detector does not have the perfect knowledge of the noise variance in real scenarios. It must cope with the so-called SNR wall (minimum value of SNR under which detection is impossible even for infinitely long samples sequence). Setting the threshold too high based on the wrong noise variance would decrease the probability of detecting the signal. If there is an x dB noise uncertainty, then the detection is impossible below [15]:

(1) $$SNRwall=10*log_{10}\left(10^{\frac{x}{10}}\;-1\right)\;\left[dB\right].$$

**Figure 1:**
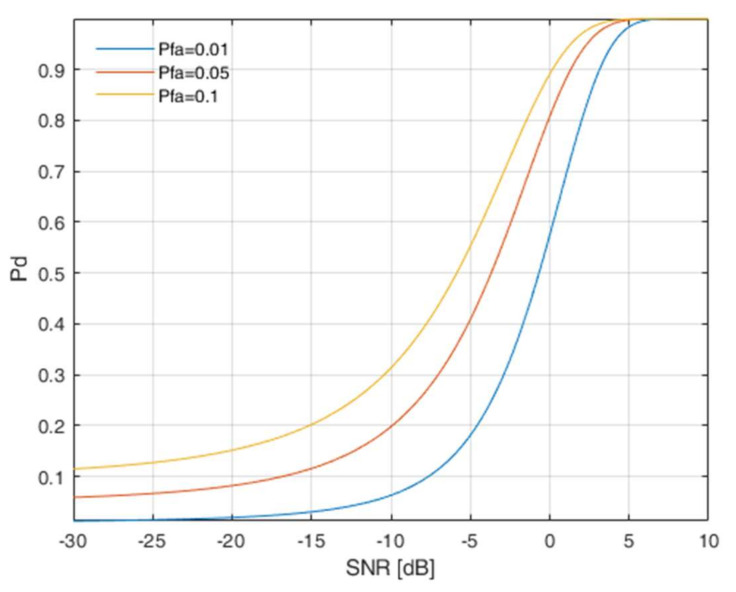
Probability of detection versus Signal to Noise Ratio of the considered ED – ENP detector.

ED-ENP is proposed because of its low complexity, good results, and flexibility. The probability of detection versus Signal to Noise Ratio of the considered detector for different values of false alarm probability is presented in Figure 1. Additionally, ED-ENP is a so-called “blind detector”. It means that it works without the knowledge of any signal parameters.

**Figure 2:**
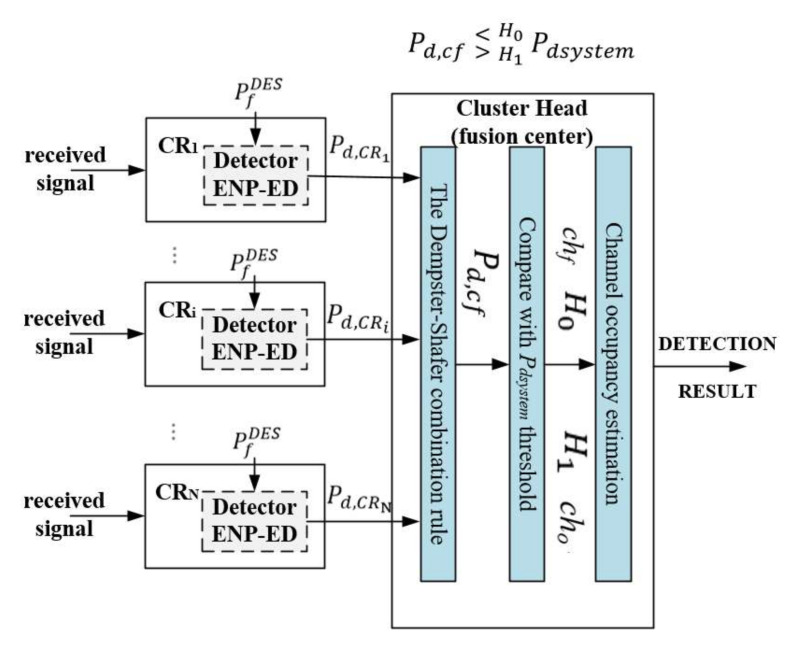
Scheme of distributed cooperative spectrum monitoring with data fusion center.

After the detection process, the local sensing results and the decisions with corresponding probabilities values ($P_{d}$ and $P_{fa}$) are transmitted to the data fusion center located in the CH in the proposed cooperative sensing solution (Figure 2). At the CH containing the fusion center, the final decision is made by comparing cooperative detection probability (fusion result $P_{d,cf}$) with Pdsystem ; Pd,cf  >< H1H0Pdsystem. $P_{dsystem}$ detection probability threshold is set by the adopted policy of radio network operation (system administrator or supervisory unit), for which a decision on radio resource occupancy is made. In other words, each CR node provides different probabilities of signal detection $P_{d}$. The CH takes a final decision whether the signal is present on the observed band or not. Based on the sensing information provided as an output: hard decision and soft decision algorithms may be used [26,27]:

- Hard decision cooperative strategies: the fusion rule combines the decisions (“hard” in each node participating in collaboration) from all the nodes. The most popular hard fusion rules are AND, OR, and majority rules. Other techniques can be based on weighted-combining strategies. The OR rule makes radio signal is present when the local detection probability in at least one node exceeds the $P_{dsystem}$ Otherwise, the second specific case of the m-out-of-K rule is the use of the logical AND operator. For this rule, no radio signal is present when the local detection probability in at least one node does not exceed the $P_{dsystem}$. The AND method minimizes the value of false alarm probability at the cost of detection probability, and the OR method maximizes detection probability at the cost of false alarm. Thus, they represent two extreme values of probability when assessing detection quality. Therefore, it seems reasonable to propose a rule that will have the advantages of OR and AND rules while excluding their disadvantages.
- Soft decision cooperative strategies imply a higher computational complexity of the fusion technique and increase the amount of information that must be exchanged among the radio nodes. Therefore, its adoption must be carefully evaluated considering the trade-off between the performance improvements and complexity increase.
- The CH must specify what kind of collaborative approach shall be used to exploit cooperative strategies.
- Fusion result is then compared to $P_{dsystem}$—system threshold of detection probability for channel occupation estimation. The final decision might be made according to different decision strategies.

### 2.2. Data Fusion Based on Dempster–Shafer Theory

The cooperative monitoring strategy allows the aggregation of monitoring information coming from several nodes. It is proved that collaborative approaches provide many benefits for monitoring purposes, such as better performances and spatial diversity. Cooperative techniques can be classified mainly into two groups, based on the monitoring information provided as output: hard decision and soft decision algorithms [26,27].

The proposed solution is based on Dempster–Shafer’s theory (DST). A complete description of this work presented by Glenn Shafer one can find in [28]. This theory is based on the use of functions defined on the power set $2^{\Theta \;}$(the set of all the subsets of Θ), where $\Theta =\;\left\{\theta_{1},\;\theta_{2},\;\theta_{3\;},\ldots \;\theta_{n}\right\}\;$ is the set of considered elements, whereas the probabilities are defined only on Θ. A mass function *m* is defined by attributing the power set $2^{\Theta \;}$ onto the range $\left[0,1\right]$ by:

(2) $$\sum_{X\in 2^{\Theta }}m\left(X\right)=1,\;m\left(\emptyset \right)=0.$$

The element X  of $2^{\Theta \;}$, such as $m\left(X\right)\;>\;0,$ is called a focal element. A mass function where Θ is a focal element is called a non-dogmatic mass function. The Dempster–Shafer (D-S) combination rule [29] for H hypothesis from  n sensors is the normalized conjunctive combination rule given for basic belief assignments $m_{1},m_{2},m_{3},\ldots ,m_{n}\;$ and for all $X\in 2^{\Theta }$, X≠∅ by:

(3) $$m\;\left(H\right)=m_{1}⨁m_{2}⨁\;\ldots \;m_{n}=\frac{\sum_{A_{1}\cap A_{2}\cap \ldots A_{n}=H}\prod_{i=1}^{n}m_{i}\left(A_{i}\right)}{1-\sum_{A_{1}\cap A_{2}\cap \ldots A_{n}=\emptyset }\prod_{i=1}^{n}m_{i}\left(A_{i}\right)}.$$

The detection probability in the fusion center is defined as:

(4) $$P_{d,\;cf}^{\;}=m\;\left(H_{1}\right)=m_{1}⨁m_{2}⨁\;\ldots m_{n}\left(H_{1}\right),$$

and the false alarm probability equals:

(5) $$P_{f,\;cf}^{\;}=m\;\left(H_{0}\right)=m_{1}⨁m_{2}⨁\;\ldots m_{n}\left(H_{0}\right).$$

The hypothesis $H_{1}$ means radio signal is detected, and $H_{0}$ no active radio signal is detected. The sum in Equation (3) is the so-called “conflict”. $\underset{A_{1}\cap A_{2}\cap \ldots A_{n}=\emptyset }{\sum }\overset{n}{\underset{k=1}{\prod }}m_{k}\left(A_{k}\right)$. In the proposed solution, we do not consider hypothesis others from Θ, which means Θ={$H_{1}$,$H_{0}$}, so normalization, in this case, is proper. One can find a detailed description of the above method in [30].

### 2.3. Radio Channels Utility Evaluation Algorithm Based on Machine Learning

**Figure 3:**
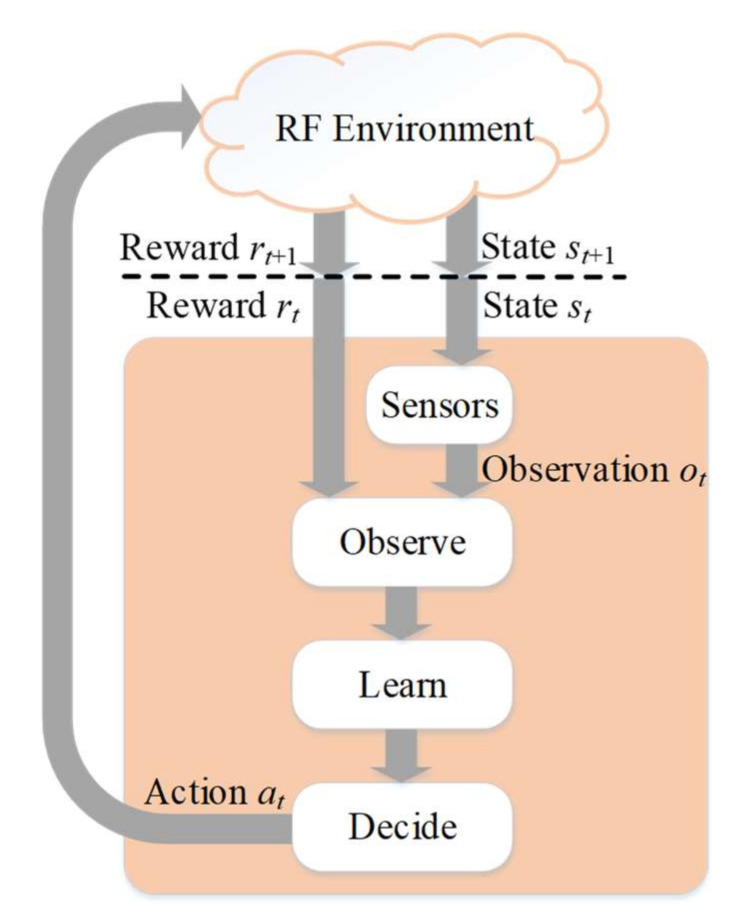
The reinforcement learning cycle, reprinted with permission from Ref. [19]. 2020 IEEE.

The central concept of this algorithm is based on the reinforcement learning cycle (Figure 3) [19]. When interacting with the environment, the learning entity makes specific actions that cause changes in the environment state and appropriate rewards. At the beginning of each cycle, the learning agent receives a full or partial observation of the current state and a reward. The next step is to learn by updating the operation policy, i.e., which actions result in the best rewards in a particular state. Finally, at the decision stage, the agent chooses an action in accordance with the modified policy. Reinforcement learning is one of the three basic types of machine learning. The other two types are supervised and unsupervised learning [31,32]. Some papers classified reinforcement learning as a subset of unsupervised learning methods [33]. Reinforcement learning methods are most suitable for spectrum monitoring and access tasks, especially in dynamically changing environments.

One of the most popular reinforcement learning methods is Q-learning. Q-learning aims to learn a policy, which tells an agent what action to take under what circumstances. It is a model-free algorithm, which does not require an environment model. Q-learning belongs to the Temporal Difference (TD) methods, and the core of this algorithm is a *Q* value update in subsequent iterations according to the following relationship:

(6) $$Q\left(s,a\right)\leftarrow Q\left(s,a\right)+\alpha \left[r+\gamma \underset{a’}{\max}Q\left(s’,a’\right)-Q\left(s,a\right)\right],$$

where *s*—current system state, *a*—action selected in the current state, *s’*—next system state, *a’*—action chosen in the next state, *Q*(*s*, *a*)—*Q* value for current state and action pair, α∈⟨0,1⟩ —learning rate, γ∈⟨0,1⟩—discount factor, and *r*—reward.

The learning rate determines the impact of new (learned) information on the current *Q* value. The discount factor determines the importance of future rewards.

If a set of environmental states cannot be defined, the *Q* values depend only on the actions. Such a case is referred to in the literature as reinforcement learning with single state [34,35] or stateless [36,37], and then the *Q* value update equation is given below:

(7) $$Q\left(a\right)\leftarrow Q\left(a\right)+\alpha \left[r-Q\left(a\right)\right].$$

The effect of defining an environment as stateless reduces the number of *Q* values estimated by the learning agent. As a result, it can also decrease the number of attempts needed to learn a mature strategy.

**Figure 4:**
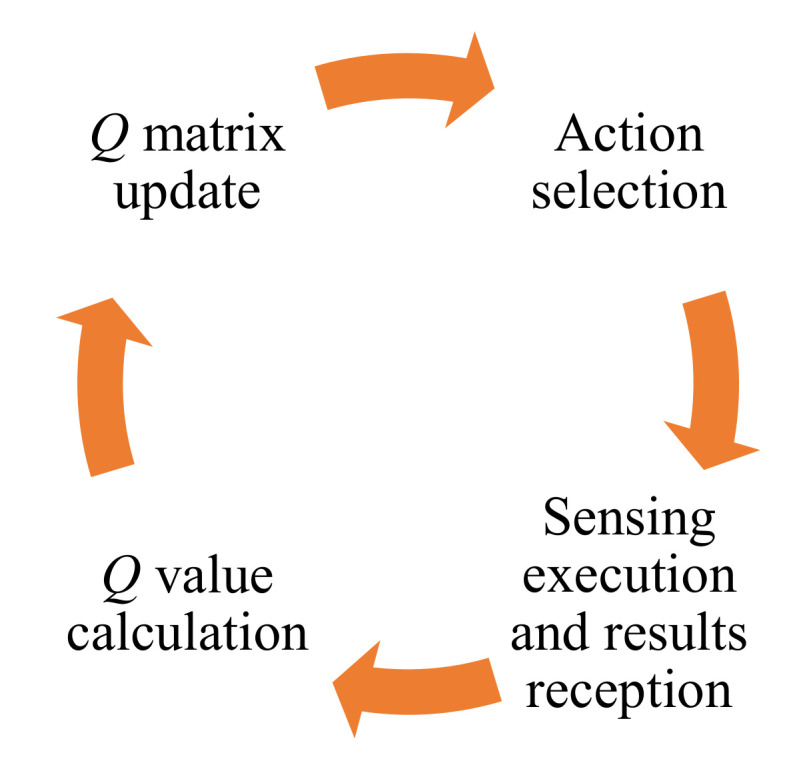
Radio channels utility evaluation algorithm—the cycle of subsequent stages.

The proposed algorithm consists of four primary stages, which form a cycle repeated during the system’s operation (Figure 4). One can find a detailed description of the above algorithm in [38].

### 2.4. Integrated Solution for Spectrum Monitoring

**Figure 5:**
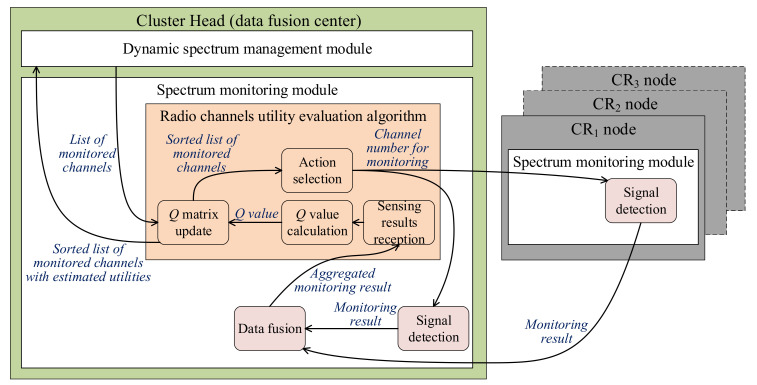
Cooperative spectrum monitoring using the centralized version of the radio channels utility evaluation algorithm.

The integration of the proposed solutions is presented in Figure 5. The authors propose to combine cooperative spectrum monitoring with data fusion based on Dempster–Shafer theory and the radio channels utility evaluation algorithm based on the machine learning method.

This solution uses the centralized radio channels utility evaluation algorithm, which calculates estimated channel qualities in one node (Cluster Head node with data fusion in Figure 5). In this case, monitoring is performed by all CR nodes on channels selected by the Cluster Head. These monitoring results are then forwarded to the data fusion module. Next, aggregated monitoring results are passed to the radio channels utility evaluation algorithm. It is proposed that the dynamic spectrum management module should provide a list of monitored channels. As a result, the spectrum monitoring module generates the sorted list of monitored channels with their estimated utilities.

## 3. Evaluation and Results

For evaluation purposes, the modules described in the previous section were implemented in the MATLAB software for scenario 1, and next, scenario 2 was implemented in the MAENA simulator.

The MAENA simulator is a high-fidelity simulator based on the OMNET++ environment. It is based on the results of the CORASMA project [39], and it enables simulation of all layers of MANET cognitive UHF and VHF waveforms, starting from the IQ-based physical layer up to the application layer. In addition, the waveform also contains cognitive solutions enabling spectrum sensing, local Dynamic Spectrum Management (DSM), and coordinated Central DSM.

The simulator also contains Radio Environment Model (REMO) responsible for propagation calculations in irregular terrain, including urban and suburban environments, using large-scale and small-scale propagation models. It also enables computations of co-site and co-vehicle effects for multi-channel radios.

### 3.1. Scenario 1

**Figure 6:**
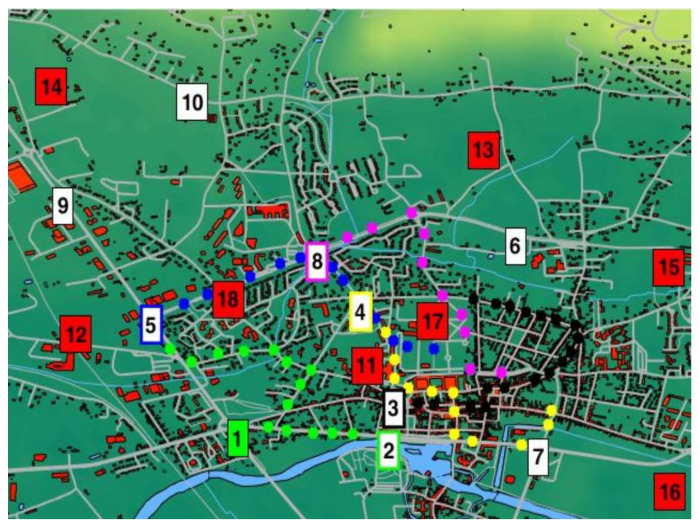
Map for nodes geolocation with their’s moving paths for the prepared scenario 1.

For all tests, authors consider a homogeneous network containing 10 CR nodes with 1 Cluster Head (CR node1—green color) and 8 Non-cooperative Nodes (NcN), representing interfering signals from primary users, legacy waveforms, or intentional jammers. Geolocation for the node’s area is presented in Figure 6. NcNs are stationary, and CRs are mobile with 5 m/s speed. Therefore, the localization area is limited to 10 km × 10 km square in the urban/suburban area. In addition, each of the CR has an ED-ENP detector with the Neyman–Pearson criterion for constant false alarm value.

**Figure 7:**
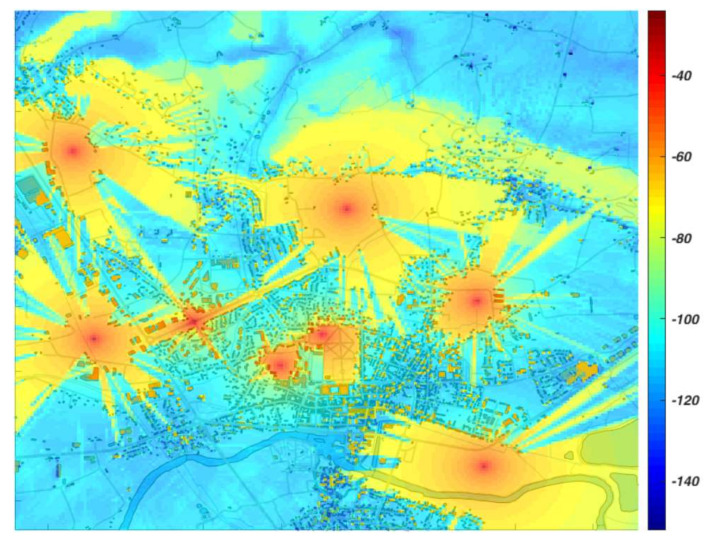
Signal power level (dBm) from NcNs for the prepared scenario 1.

For the path loss calculation, REMO [40] module is used. It contains combined propagation models that fit specific frequency bands, communications ranges, and terrain conditions. Apart from that, REMO simulates the non-ideal properties of radio transmitters and receivers. It also enables the generation of interferences coming from NcNs. An example of calculated NcN signals power in the urban area is presented in Figure 7.

Scenario parameters are as follows:

- Antenna height = 2,5 (m);
- Antenna gain = 0 (dBi);
- CR sensitivity ≈ −105 (dBm);
- NcN power = 5 (W);
- Noise type = AWGN.

**Figure 8:**
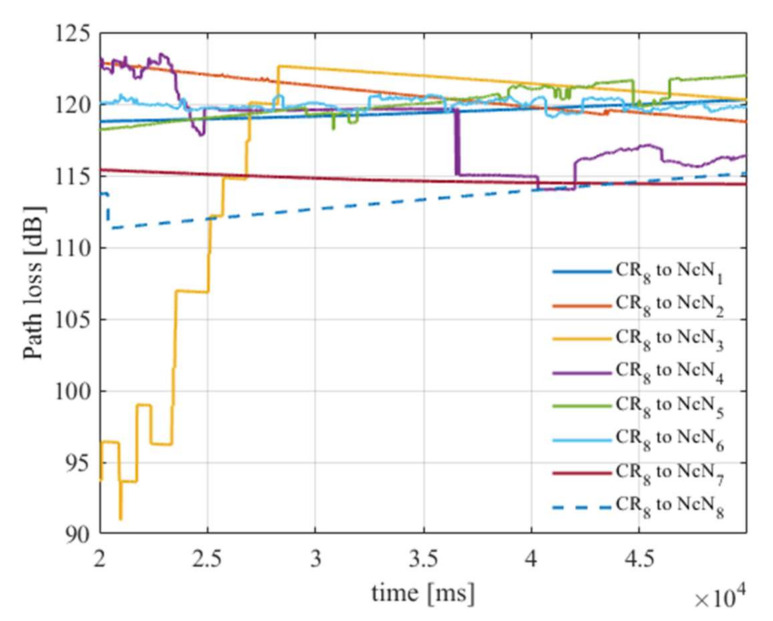
Path loss for Cognitive Radio node 8 and all NcNs from 20 s to 50 s simulation time for scenario 1.

As an example, in Figure 8, path loss for CR node number 8 and all NcNs is presented.

**Figure 9:**
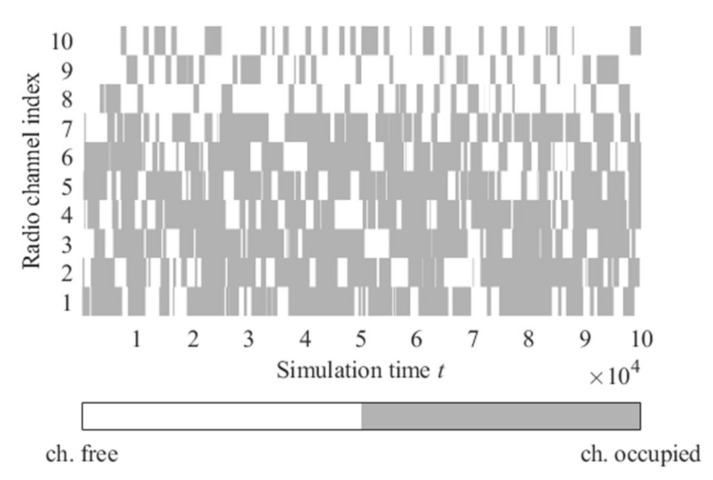
Radio channels activities for the prepared scenario 1.

Radio channel activities (interfering signals from NcNs) are presented in Figure 9. The following parameters of the radio channels utility evaluation algorithm are used during the simulations:

- *α* = 0.5;
- *ε* = 0.3;
- A fixed seed value of the pseudorandom number generators (depending on the seed value, there may be some differences in the results);
- Algorithm period = 100 ms (time between successive algorithm iterations—updates of the *Q* value for the selected channel based on the monitoring results).

#### 3.1.1. Metrics for Evaluation

To evaluate the efficiency of the spectrum monitoring process (for all monitored channels), the metric *SOAR* (Spectrum Occupancy Awareness ratio) is proposed. This is the average value obtained from the detection rates of all monitored channels and can be written as:

(8) $$SOAR=\frac{\sum_{i=1}^{G_{o}}\left(P_{d,\;cf}\left(i\right)\;>\;P_{dsystem}\right)}{G_{o}},$$

where $G_{o}$—number of occupied states from channels activities, $P_{d,\;cf}$—detection probability in the fusion center, $P_{dsystem}$—system threshold of detection probability for channel occupation estimation, $SOAR\in \left(0,1\right)$.

The utility ratio is a metric used to evaluate the usefulness of selected channels. It can be calculated for every radio channel using the following formula:

(9) $$Utl=\;\frac{N_{f}}{T},$$

where $N_{f}$—the number of free (idle) channel samples, T—the number of all channel samples (scenario duration).

#### 3.1.2. Results

**Figure 10:**
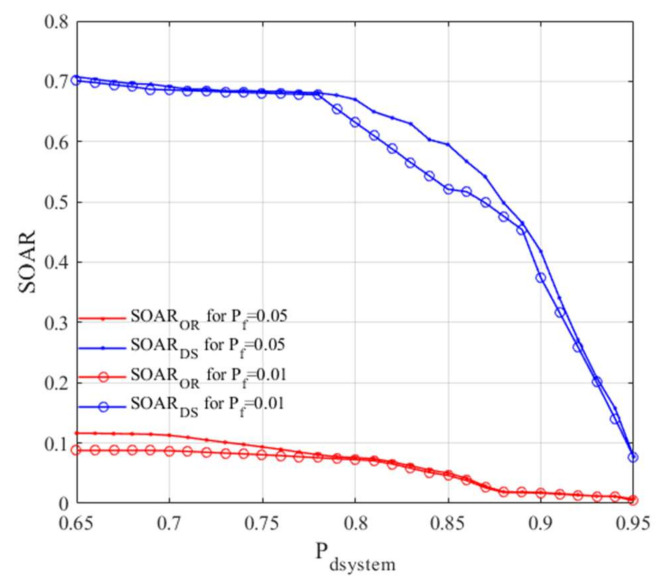
Spectrum Occupancy Awareness Ratio for different system probability thresholds and combination rules for scenario 1.

Results for the proposed data fusion with comparison to OR fusion method are presented in Figure 10. As one can see, the proposed solution outperforms the classical OR method, commonly used in many applications.

**Figure 11:**
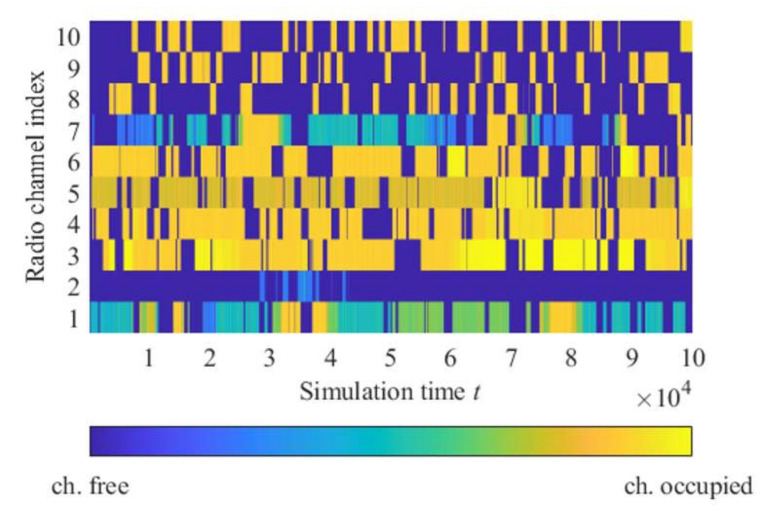
Aggregated monitoring results for all channels.

Results (soft-decision/detection probability) from the monitoring block for each channel from all CR nodes delivered to the fusion center are presented in Figure 11.

The sensing procedures indicate a small radio activity on channel no. 2 due to geolocation conditions. For such a situation, channel no. 2 is almost always available. In this case, our solution would always indicate channel no. 2 as the best channel—characterized by the least occupancy. Therefore, to enforce spectrum sharing in all available channels, a modification of the scenario is proposed to exclude channel no. 2 from assigned resources.

**Figure 12:**
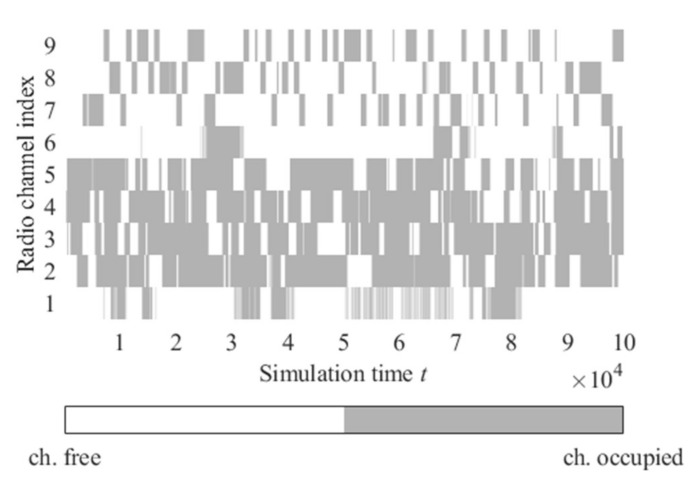
Aggregated sensing results for $P_{dsystem}$ = 0.65.

In the fusion center, an aggregation of monitoring information coming from all nodes using data fusion based on Dempster–Shafer’s theory (Section 2.2) is carried out. Then, depending on the system probability detection threshold, the decision of spectrum resource (channel) occupation is performed, like, for example, in Figure 12. Those sensing results are input data for the machine learning algorithm. It is assumed that if the sensing result indicates that a particular channel is free (idle), its occupation will not cause a collision with other spectrum users. Therefore, even if there are any radio activities on this channel, it could not affect the network’s operation, e.g., channel no. 2 in Figure 11.

**Figure 13:**
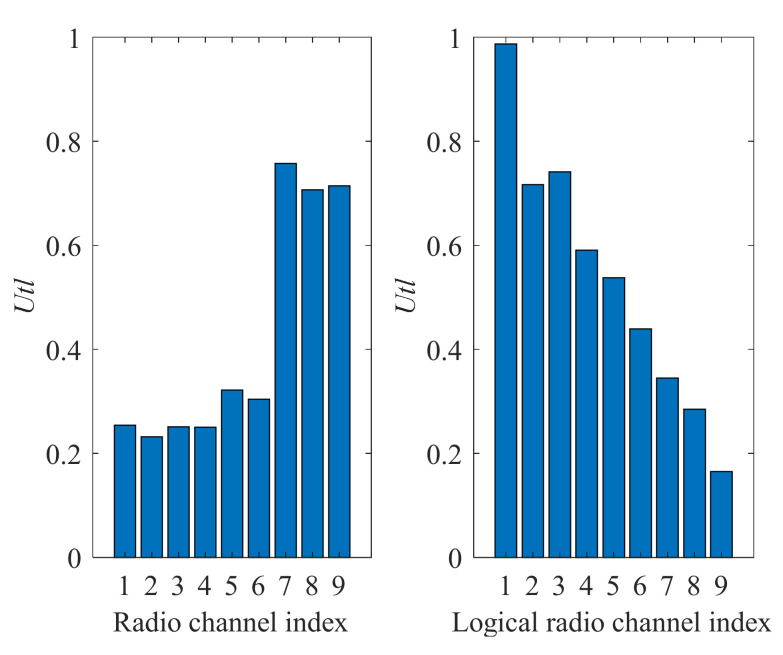
Utility ratio (*Utl*) values for physical radio channels (on the **left**) and logical radio channels selected by the proposed algorithm (on the **right**).

**Figure 14:**
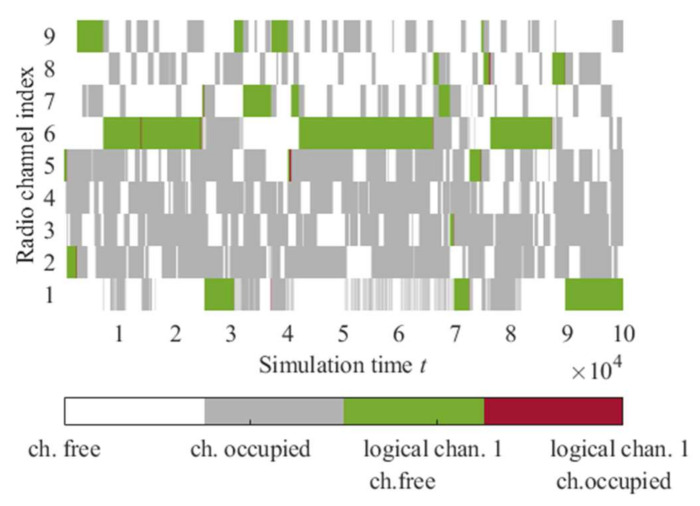
Radio channels activities with logical channel one.

Figure 13 presents the utility ratio (*Utl*) values for physical and logical radio channels selected by the proposed algorithm. Physical radio channels are associated with assigned radio frequency bands. In contrast, logical channels are sequences composed of these physical channels (Figure 14) and are associated with the *Q* matrix prepared by the radio channels utility evaluation algorithm. This matrix contains *Q* values (quality metric) for all physical channels. After each sensing execution and results reception, the *Q* value for a given physical channel is recalculated, and the list is updated (as shown in Figure 4). Then the list is sorted to get the best physical channels. The logical channels are related to the indexes in this matrix, i.e., logical channel 1 indicates the physical channels in the best position (index no. 1) on the *Q* list in successive moments. An example of such a logical channel is presented in Figure 14. As can be seen, the logical channel with index one is characterized by much better *Utl* values than physical channels, which provides gain compared to the operation on the static physical radio channels. The changes in this logical channel over time are shown in Figure 14. Right decisions (free channel selections) are marked in green, whereas bad decisions (occupied channel selections)—in red.

### 3.2. Scenario 2

**Figure 15:**
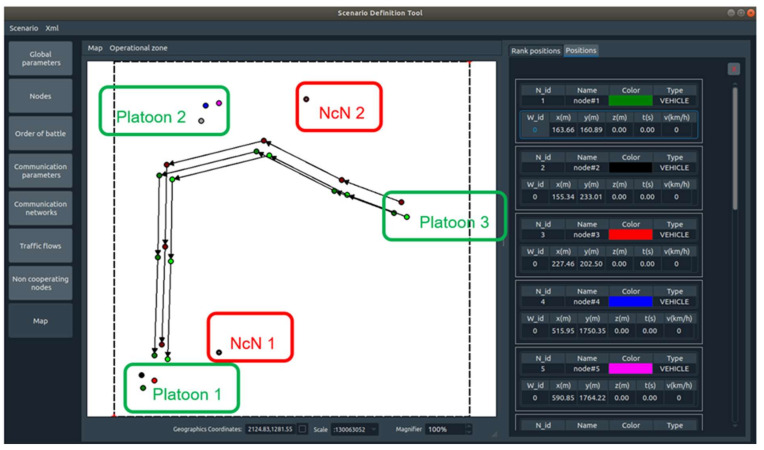
Map for scenario 2.

To verify and evaluate the proper operation of the proposed solution in the Cognitive Radio Network (MAENA Simulator), scenario 2 was prepared (Figure 15). This is the situation for three platoons. Each platoon contains three radio nodes—two Communication Nodes and one Cluster Head. Platoon 1 and Platoon 2 are static, while Platoon 3 is mobile. The path for the Platoon 3 movement is presented in Figure 15. All radio nodes use the basic UHF waveform based on CORASMA waveform [41], enabling the creation of self-organizing radio networks, grouping nodes into clusters, the election of cluster heads (managing the nodes belonging to the cluster), and gateways responsible for inter-cluster communications. Next, cluster graph coloring is performed to use the assigned spectral resources efficiently. Compared to the CORASMA waveform, the reference waveform has several additional features: frequency hopping, management of retransmissions, and optimized routing procedures. This reference waveform is extended with additional functions by solutions proposed in this article. Scenario 2 is run with different policies/strategies for Sensing, Data Fusion, and the radio channels utility evaluation algorithm to obtain different network behavior, according to the mission goals and existing restrictions. The defined policies should enable, e.g., maximization of waveform data rate available for the users, or battery life extension, minimization of transmission detection probability, or increased immunity to interference in a variable spectral situation.

Two NcNs are also defined. NcN1 operates on frequency 227.625 MHz, from 5 to 7 s scenario time—it is introduced for intentional jamming or primary user emulation; please refer to Figure 15.

#### 3.2.1. Metric for Evaluation

The metric ANSE (Average Network Spectrum Efficiency) was proposed to evaluate the elaborated solution efficiency compared to the non-cognitive network and consider bitrate throughput and consumed spectrum resources [41]. However, before defining the ANSE, let us specify the NSE (Network Spectrum Efficiency) metric for a single network:

(10) $$NSE_{i}=\;\frac{R_{i}}{b_{i}\cdot d_{i}},$$

where $R_{i}$—available transmission speed in each network i, $b_{i}\cdot d_{i}$—bandwidth-duration product.

Average Network Spectrum Efficiency for n number of networks can be defined as follows:

(11) $$ANSE=\;\frac{\sum_{i=1}^{n}NSE_{i}}{n}\;\left[\frac{\frac{bit}{s}}{MHz\;h}\right].$$

The implementation of the components of the above formula in the OMNET++ environment covers:

- The available transmission speed $\;R_{i}$ is replaced by the total number of transferred bits;
- $b_{i}$ is calculated as the number of used channels times the bandwidth for each channel;
- $d_{i}$ is calculated as the difference in time between the first and the last transferred bit in each network.

As a consequence of the change from transmission speed to the total number of bits, the unit of ANSE is a bit/(MHz h) instead of bit/s/(MHz h).

#### 3.2.2. Results

**Figure 16:**
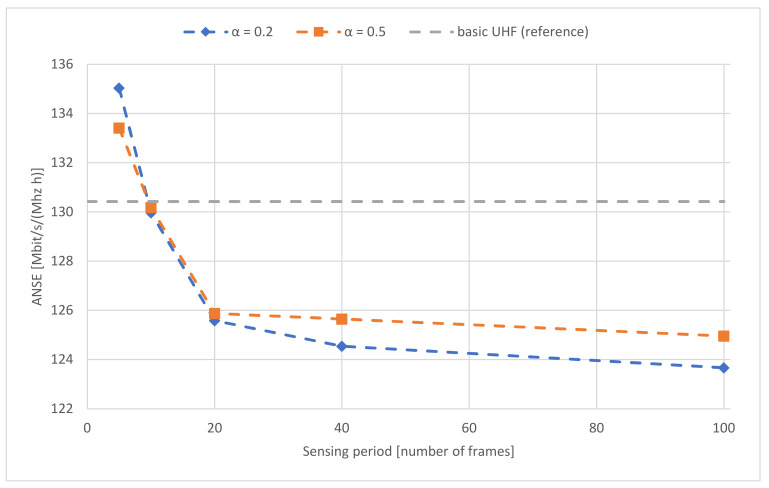
Average Network Spectrum Efficiency vs. different sensing periods for scenario 2.

ANSE results for scenario 2 are presented in Figure 16. Tests were performed for different parameters values: learning rate α (0.2 and 0.5) and sensing period (time between successive detection scaled in frames). As a reference, results for the basic UHF waveform are also shown.

Based on the result from spectrum monitoring realized using centralized cooperative spectrum sensing and radio channel utility evaluation, Cluster Head has information about the channel occupation ranking list for the whole cluster. This approach allows defining required policies/strategies, using a set of parameters like sensing periodicity, type of the sensing decision (soft/hard), sensing reporting periodicity, learning rate (determines the impact of learned information on the current Q value), etc. Depending on the strategy of the network operation an example ones are:

- Goal 1: keep the network alive in the very jammed/interfered radio environment (pros.—the network has the most current situation awareness; cons.—consuming a significant number of network resources for spectrum monitoring).
- Goal 2: minimize data consumption for the situation awareness building (pros.—consuming a small number of network resources for spectrum monitoring; cons.—the network does not have the most current situation awareness).
- Goal 3: a compromise between having a current situation awareness and data consumption for the spectrum monitoring.

The percentage of the consumed network resources for the spectrum monitoring process (channel occupation estimation) for the above strategies is presented in Table 1.

The proposed basic strategies do not exhaust the possible solutions. Depending on the desired operation of the network, the parameters of the algorithms should be appropriately selected. For example, if the network is supposed to operate at all costs in a highly disturbed radio environment, the proposed solution is Goal 1.

## 4. Conclusions

This paper presents an efficient cooperative spectrum monitoring methodology based on a group of radios’ with energy detection. The results, devoted to specific radio channels, are then transmitted to the fusion center, which uses evidence theory to increase the signal detection efficiency and provide a low false alarms ratio. The fusion results are the input for the machine learning algorithm, evaluating specific radio channels’ utility factors.

The achieved results are promising. The authors proposed a sensing solution based on distributed cooperative spectrum monitoring with a central node (fusion center/Cluster Head) with the D-S combination rule for the decision process and reinforced Machine Learning. Simulation results show that the proposed solution increases Average Network Spectrum Efficiency. However, the gain depends on the learning curve related to the variability of frequency channel occupancy and network resources consumed for sensing.

During future works, authors plan to develop and test algorithms with a distributed version of the radio channels utility evaluation algorithm, selecting the best radio channels performed in each CR node, to enable limitation of the signaling overhead.

## Figures and Tables

**Table 1:** Percentage of the consumed resources for different strategies.

Strategy Name	Sensing Period [Frames]	Percentage of the Consumed Resources for Spectrum Monitoring (%)
Goal 1	5	20
Goal 2	100	1
Goal 3	20	5

## References

[1] Arslan H. (2007). Cognitive Radio, Software Defined Radio, and Adaptive Wireless Systems.

[2] Marin J., Turunen M., Bernhardt M., Riihonen T. Self-interference Cancelation Performance in Full-Duplex Jamming and Spectrum Monitoring. Proceedings of the 2021 International Conference on Military Communication and Information Systems (ICMCIS).

[3] Liu P., Qi W., Yuan E., Wei L., Zhao Y. (2017). Full-Duplex Cooperative Sensing for Spectrum-Heterogeneous Cognitive Radio Networks. Sensors.

[4] Mehdawi M., Riley N., Fanan A., Bentaher O. Proposed System Model for Wideband Cooperative Spectrum Sensing with Multi-Bit Hard Decision using Two-Stage Adaptive Sensing. Proceedings of the 2019 27th Telecommunications Forum (TELFOR).

[5] Kartlak H., Odabasioglu N., Akan A. Optimum relay selection for cooperative spectrum sensing and transmission in cognitive networks. Proceedings of the 2014 22nd European Signal Processing Conference (EUSIPCO).

[6] Mahapatra S.D., Sharan S.N. Effect of Sensing Duration Optimization in Cooperative Spectrum Sensing Game. Proceedings of the 2018 7th International Conference on Reliability, Infocom Technologies and Optimization (Trends and Future Directions) (ICRITO).

[7] Szmit G., Dołowski J., Łopatka J. Distributed channel selection for hierarchical cognitive radio networks. Proceedings of the MILCOM 2015—2015 IEEE Military Communications Conference.

[8] Wu K., Jiang H., Tellambura C. (2021). Cooperative Sensing with Heterogeneous Spectrum Availability in Cognitive Radio. IEEE Transactions on Cognitive Communications and Networking.

[9] Baek M.K., Kim J.Y. Effective signal detection using cooperative spectrum sensing in cognitive radio systems. Proceedings of the 2009 11th International Conference on Advanced Communication Technology.

[10] Nasser A., Al Haj Hassan H., Abou Chaaya J., Mansour A., Yao K.-C. (2021). Spectrum Sensing for Cognitive Radio: Recent Advances and Future Challenge. Sensors.

[11] Valadão M.D.M., Amoedo D., Costa A., Carvalho C., Sabino W. (2021). Deep Cooperative Spectrum Sensing Based on Residual Neural Network Using Feature Extraction and Random Forest Classifier. Sensors.

[12] Yücek T., Arslan H. (2009). A survey of spectrum sensing algorithms for cognitive radio applications. IEEE Commun. Surv. Tutor..

[13] Qu H., Xu X., Zhao Yan F., Wang W. (2020). A Robust Hyperbolic Tangent-Based Energy Detector with Gaussian and Non-Gaussian Noise Environments in Cognitive Radio System. IEEE Syst. J..

[14] Mariani A., Giorgetti A., Chiani A. (2011). Effects of noise power estimation on energy detection for cognitive radio applications. IEEE Trans. Commun..

[15] Mariani A., Giorgetti A., Chiani A. SNR wall for energy detection with noise power estimation. Proceedings of the IEEE International Conference on Communications.

[16] Akyildiz I.F., Lo B.F., Balakrishnan R. (2011). Cooperative spectrum sensing in cognitive radio networks: A survey. Phys. Commun..

[17] Ma Y., Liu J., Gao Y. Cooperative spectrum sensing based on the compressed sensing. Proceedings of the 2015 IEEE 14th International Conference on Cognitive Informatics & Cognitive Computing (ICCI*CC).

[18] Skokowski P., Łopatka J., Malon K. Evidence Theory Based Data Fusion for Centralized Cooperative Spectrum Sensing in Mobile Ad-hoc Networks. Proceedings of the 2020 Baltic URSI Symposium (URSI).

[19] Malon K., Łopatka J., Skokowski P. Q-learning Based Radio Channels Utility Evaluation Algorithm for the Local Dynamic Spectrum Management in Mobile Ad-hoc Networks. Proceedings of the 2020 Baltic URSI Symposium (URSI).

[20] Du R., Liu F., Zhao Q. Adaptive cooperative spectrum sensing based on multiple measurement vectors. Proceedings of the 2017 29th Chinese Control and Decision Conference (CCDC).

[21] Malon K., Skokowski P., Lopatka J. (2018). Optimization of wireless sensor network deployment for electromagnetic situation monitoring. Int. J. Microw. Wirel. Technol..

[22] Malon K., Skokowski P., Lopatka J. Optimization of the MANET topology in urban area using redundant relay points. Proceedings of the International Conference on Military Communications and Information Systems (ICMCIS).

[23] Digham F., Alouini M.S., Simon M.K. (2007). On the Energy Detection of Unknown Signals over Fading Channels. IEEE Trans. Commun..

[24] Sonnenschein A., Fishman P.M. (1992). Radiometric detection of spread-spectrum signals in noise of uncertain power. IEEE Trans. Aerosp. Electron. Svst..

[25] Neyman J., Pearson E.S. (1933). On the Problem of the Most Efficient Tests of Statistical Hypotheses. Philos. Trans. R. Soc..

[26] Bedworth M.D. (2002). The Fusion of Decisions for Distributed Recognition: Hard Decision Fusion and Soft Decision Fusion. Multisensor Fusion.

[27] Hall D.L. (2002). The Implementation of Data Fusion Systems. Multisensor Fusion.

[28] Shafer G.A. (1976). Mathematical Theory of Evidence.

[29] Smarandache F., Dezert J. (2004). Advances and Applications of DSmT for Information Fusion.

[30] Skokowski P. (2021). Electromagnetic Situation Awareness Building in Ad-Hoc Networks with Cognitive Nodes.

[31] Biglieri E., Goldsmith A.J., Greenstein L.J., Mandayam N.B., Poor H.V. (2013). Principles of Cognitive Radio.

[32] Doyle L. (2009). Essentials of Cognitive Radio.

[33] Bkassiny M., Li Y., Jayaweera S.K. (2013). A survey on machine-learning techniques in cognitive radios. IEEE Commun. Surv. Tutor..

[34] Sutton R.S., Barto A.G. (2018). Reinforcement Learning: An Introduction.

[35] Yau K.L.A., Komisarczuk P., Teal P.D. Applications of reinforcement learning to cognitive radio networks. Proceedings of the IEEE International Conference on Communications Workshops.

[36] Claus C., Boutilier C. The dynamics of reinforcement learning in cooperative multiagent systems. Proceedings of the Fifteenth National/Tenth Conference on Artificial Intelligence/Innovative Applications of Artificial Intelligence.

[37] Morozs N., Clarke T., Grace D. (2016). Distributed heuristically accelerated Q-learning for robust cognitive spectrum management in LTE cellular systems. IEEE Trans. Mob. Comput..

[38] Malon K. (2021). Dynamic Spectrum Access in Ad-Hoc Radio Networks with Cognitive Nodes.

[39] Rose L., Massin R., Vijayandran L., Debbah M., Le Martret C.J. CORASMA Program on Cognitive Radio for Tactical Networks: High Fidelity Simulator and First Results on Dynamic Frequency Allocation. Proceedings of the MILCOM 2013—2013 IEEE Military Communications Conference.

[40] Malon K., Skokowski P., Marszalek P., Kelner J.M., Lopatka J. Cognitive Manager for Hierarchical Cluster Networks Based on Multi-Stage Machine Method. Proceedings of the IEEE Military Communications Conference MILCOM.

[41] Frequency Management Group Range Commanders Council (2014). Document 707-14 Spectrum Management Metrics Standards.

